# Cytokine expression patterns in hospitalized children with Bordetella pertussis, Rhinovirus or co-infection

**DOI:** 10.1038/s41598-021-89538-0

**Published:** 2021-05-26

**Authors:** Elisabetta Pandolfi, Nadia Panera, Anna Alisi, Emanuela Carloni, Luisa Russo, Ilaria Campagna, Caterina Rizzo, Carlo Concato, Giulia Linardos, Livia Piccioni, Sally Jackson, Alberto Villani, Fabio Midulla, Alberto E. Tozzi

**Affiliations:** 1 Multifactorial Disease and Complex Phenotype Research Area, Bambino Gesù Children’s Hospital (IRCCS), Piazza S. Onofrio, 4, 00165 Rome, Italy; 2 Research Unit of Molecular Genetics of Complex Phenotypes, Bambino Gesù Children’s Hospital (IRCCS), Rome, Italy; 3 Virology Unit, Laboratory Department, Bambino Gesù Children’s Hospital (IRCCS), Rome, Italy; 4 Department of Pediatrics, Bambino Gesù Children’s Hospital (IRCCS), Rome, Italy; 5 Department of Pediatrics, Sapienza University, Rome, Italy

**Keywords:** Microbiology, Medical research

## Abstract

Mechanisms of interaction between Bordetella pertussis and other viral agents are yet to be fully explored. We studied the inflammatory cytokine expression patterns among children with both viral-bacterial infections. Nasopharyngeal aspirate (NPA) samples were taken from children, aged < 1 year, positive for Rhinovirus, Bordetella pertussis and for Rhinovirus and Bordetella pertussis. Forty cytokines were evaluated in NPA by using human cytokine protein arrays and a quantitative analysis was performed on significantly altered cytokines. Forty cytokines were evaluated in NPA by using human cytokine protein arrays and a quantitative analysis was performed on significantly altered cytokines. Our results show that co-infections display a different inflammatory pattern compared to single infections, suggesting that a chronic inflammation caused by one of the two pathogens could be the trigger for exacerbation in co-infections.

## Introduction

Despite a high childhood vaccination rate, *Bordetella pertussis* (Bp) outbreaks occur periodically and Whooping cough–also known as Pertussis–remains a public health problem particularly for infants^1–3^ and children, for whom it remains one of the most fatal infections^4^.

Pertussis continues to circulate and outbreaks occurs worldwide, also in industrialized countries (particularly California and Australia) where immunization uptake for pertussis is high since a long time have underlined that current strategies for preventing pertussis are insufficient to control the disease. One possible explanation of this resurgence is that the currently available vaccines against pertussis protect incompletely and for a limited time. This leads to a high morbidity and mortality in infants, under 3 months of age, too young to benefit from immunization, with severe infection requiring hospitalization, including an intensive care setting. Beyond the development of more efficacious vaccines that induce protection in the long term, alternative integrated strategies should be therefore considered for limiting the burden of pertussis^2,5,6^.

Pertussis may be recognised by its typical clinical presentation that includes apnoea, paroxysmal cough, inspiratory whoop, and post-cough vomiting. However, symptoms may vary with age and immunisation status, thus the clinical diagnosis of pertussis may be missed in older individuals and those who are immunised, as their clinical manifestations are milder^7^. For this reason, standardised and specific laboratory confirmation is necessary to avoid misdiagnosis, even when Bp infection can be reasonably suspected from typical clinical symptom presentation^8^.

Co-infections with respiratory viruses frequently occur in infants^9^ and may present clinically similarly to pertussis^10^.

The co-circulation dynamics and interactions between different pathogens in the same host and ecological niche can be complex. Simultaneous infection with multiple pathogen species is particularly frequent in the upper respiratory tract, where exposure to microbial species is common and interactions between pathogens, which are often host-mediated, are particularly relevant[^11^]. One of the best-known examples of interspecies interactions in the respiratory tract is the 1918 influenza pandemic, during which 95% of mortality was attributed to bacterial co-infection^12,13^. Bp and respiratory virus co-infection is often found in hospitalised infants younger than 6 months, although it is not clearly understood if the co-infection is associated with specific pathogenetic mechanisms^14,15^. It has been hypothesised that Bp could predispose the respiratory tract to viral infection through sensitising the host to a respiratory pathogen through suppression of the innate immune response^16^. Conversely, respiratory and influenza viruses may support bacterial co-infection through the promotion of the activation of type I interferons (IFNs) and release of pro-inflammatory cytokines^16–18^.

As the presence of a pathogen does not necessarily correspond to clinical outcomes, the study of the host response in co-infections may provide additional insights into pathogenic mechanisms. Identifying biomarkers of co-infection is desirable as the correct interpretation of clinical symptoms may be relevant to decision-making, and to the precise estimation of the burden of pertussis.

In previous work, we observed that human Rhinovirus (RV) is the most frequent respiratory virus associated with Bp in infants less than 6 months of age (15). Little is known about the pathogenic synergies, host response, and pro-inflammatory patterns in Bp and RV co-infections. Therefore, in order to gain additional insights into pathogenic mechanisms, we performed a study aimed at describing and comparing the clinical patterns and nasopharyngeal cytokine profiles in children < 12 months old with single or mixed Bp and RV infections.

## Results

### General and clinical characteristics of patients by type of infection

Fifty-eight children were enrolled, among whom 24 (41.4%) had an RT-PCR positive for pertussis, 14 (24%) were RT-PCR positive for rhinovirus and negative for pertussis, and 20 (34.5%) had both pertussis-rhinovirus infection. The socio-demographic characteristics of patients enrolled by type of infection are described in Table 1. Patients with co-infection (Bp + RV) were slightly older and tended to have a higher birth weight compared with children with RV or Bp alone. Clinical characteristics of patients by type of infection are reported in Table 2. Cough duration was higher in patients presenting with co-infection and pertussis. Co-infections also had a value of lymphocytes and white cell count that was higher than the maximum value expected for age. No difference was found for complications among the three groups. Antibiotic treatment was given most frequently in patients with co-infection and in patients with pertussis, and it was a macrolide in 67% and 62.5% of cases respectively.

**Table 1:** Socio-demographic characteristics of patients enrolled by type of infection.

	Pertussis	Rhinovirus	Co-infections	Total	*P*
Male (n, %)	13 (54.2)	9 (64.3)	12 (60)	34 (58.6)	0.82
Age in months (mean, SD)	2.0 (1.5)	1.8 (1.3)	2.7 (1.4)	2.2 (1.5)	0.10
Caucasian (n, %)	23 (95.8)	14 (100)	20 (100)	57 (98.2)	0.49
Gestational age in weeks (mean, SD)	38.3 (2.4)	36.9 (3.7)	38.4 (1.9)	38.0 (2.7)	0.61
Premature birth (n, %)	3 (12.5)	5 (35.7)	3 (15)	11 (19)	0.22
Birth weight in kg (mean, SD)	3.2 (0.7)	2.8 (0.8)	3.3 (0.6)	3.1 (0.7)	0.18
Caesarean birth (n, %)	8 (33.3)	10 (71.4)	11 (55)	29 (50)	0.07
Chronic diseases (n, %)	1 (4.8)	2 (18.2)	0	3 (5.9)	0.16
Exclusive breastfeeding (days)	31.7 (35.4)	21.6 (29.6)	53.7 (63.4)	37.1 (47.3)	0.40
Employed mother (n, %)	16 (72.7)	8 (72.7)	10 (52.6)	34 (65.4)	0.39
Employed father (n, %)	20 (90.9)	10 (90.9)	19 (100)	49 (94.2)	0.42
Mother with university degree (n, %)	4 (23.5)	2 (18.2)	6 (30)	12 (25.5)	0.76
Father with university degree (n, %)	6 (37.5)	4 (36.4)	4 (21)	14 (30.4)	0.55
No of Households (mean, SD)	3.7 (0.6)	4.2 (0.8)	4.2 (1.3)	4.0 (1.0)	0.20
At least one sibling (n, %)	14 (58.3)	12 (85.7)	14 (70)	40 (69)	0.21
Smoker mother (n, %)	1 (5.6)	4 (36.4)	3 (15.8)	8 (16.7)	0.11

Data are reported as number and percentage, or as mean value and standard deviation (SD) reported in round brackets. Statistical significance was evaluated by Chi-square test or Fisher’s exact test for proportions and Kruskal–Wallis test for means.

**Table 2:** Clinical characteristics of patients by type of infection.

Symptom	Pertussis	Rhinovirus	Co-infections	*P*
Cough (n, %)	22 (91.7)	12 (85.7)	20 (100)	0.21
Paroxysmal cough (n, %)	21 (87.5)	9 (64.3)	17 (85)	0.22
Cough duration in days (mean , SD)	10.6 (6.9)	3.38 (4.23)	11.6 (5.4)	< 0.001
Apnoea (n, %)	17 (70.8)	6 (42.9)	13 (65)	0.22
Cyanosis (n, %)	18 (75)	6 (42.9)	13 (65)	0.14
Whooping (n, %)	18 (75)	4 (28.6)	11 (55)	0.02
Post-tussive vomiting (n, %)	14 (58.3)	4 (28.6)	12 (60)	0.16
Fever (n, %)	4 (16.7)	6 (42.9)	3 (15)	0.13
White cell count > max for age	12 (52.2)	2 (18.2)	15 (75)	0.01
Lymphocytes value > 50%	19 (82.6)	4 (36.4)	19 (95)	0.001
White cell count > max for age and Lymphocytes value > 50% (%)	9 (39.1)	0	14(70)	< 0.001
Conjunctival haemorrhage (n, %)	4 (16.7)	0	2 (10)	0.36
Petechiae (n, %)	2 (8.3)	0	3 (15.8)	0.35
Length of hospital stay (mean, SD)	8.9 (5.4)	11.9 (26.0)	9.4 (5.5)	0.07
Antibiotic treatment before admission (n, %)	7 (30.4)	2 (16.7)	12 (63.2)	0.02
Admission in PICU (n, %)	2 (8.3)	1 (7.7)	0	0.44

### Evaluation of the pattern of expression of a panel of inflammatory mediators in children with Bp, RV infection or co-infection

**Figure 1:**
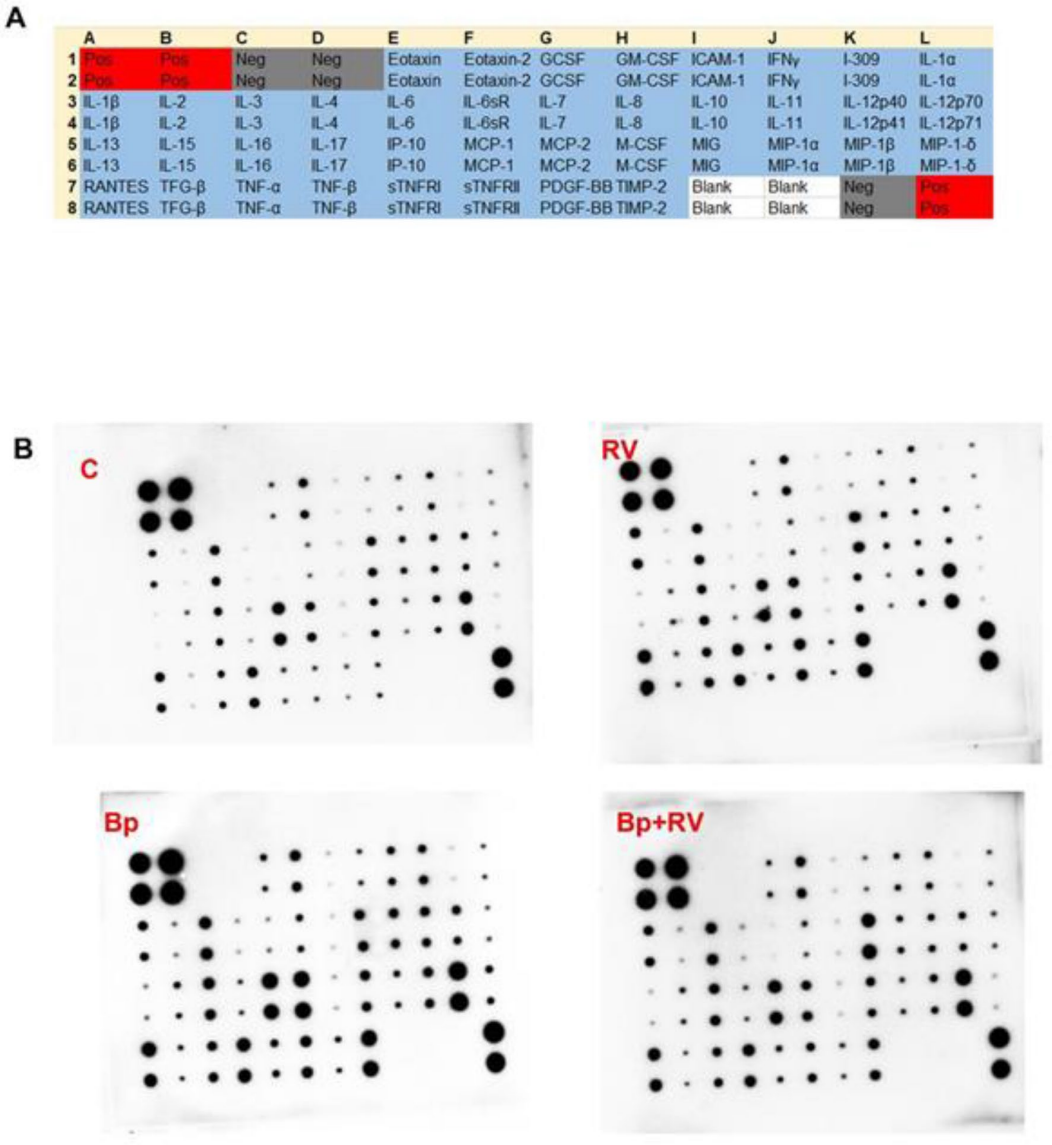
Inflammatory pattern by antibody array. (**A**) Layout of the antibodies as spotted on the membrane. The abbreviated names for 40 different cytokine probes are reported. (**B**) Representative photographs of cytokine arrays in which each cytokine is represented by duplicate spots on a single membrane.

In order to identify potential inflammatory mediators displaying a different pattern of expression in the different group of infected patients, we evaluated a panel of 40 inflammatory molecules (Fig. 1A) in 6 control samples, and in NPA from 13 children positive for RV; 11 children positive for Bp; 16 children positive for both Bp + RV.

**Figure 2:**
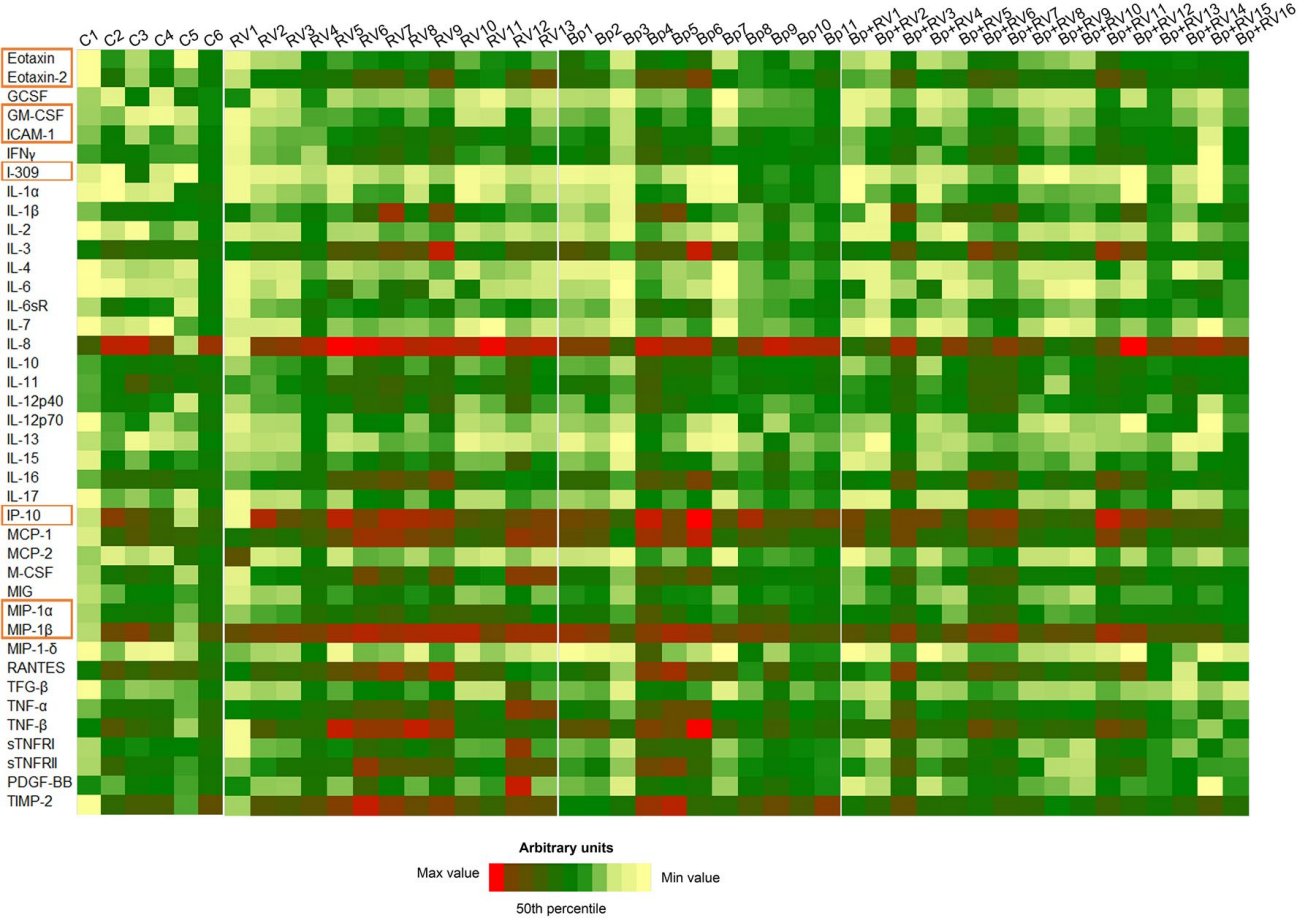
Identification of pattern expression of inflammatory proteins by antibody arrays. Heatmap representation of the mean of semi-quantitative expression of the 40 inflammatory proteins in controls, RV patients, Bp patients**,** and Bp + RV patients.

**Figure 3:**
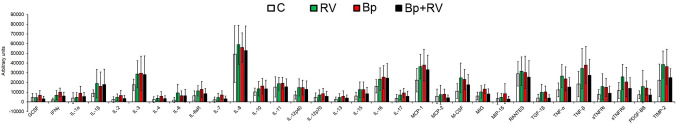
Pattern of undifferentiated inflammatory proteins in the screening cohort. The histogram reported the mean ± SD of semi-quantitative expression in controls (C), RV patients, Bp patients, and Bp + RV patients of undifferentiated inflammatory molecules screened by antibody-array.

**Figure 4:**
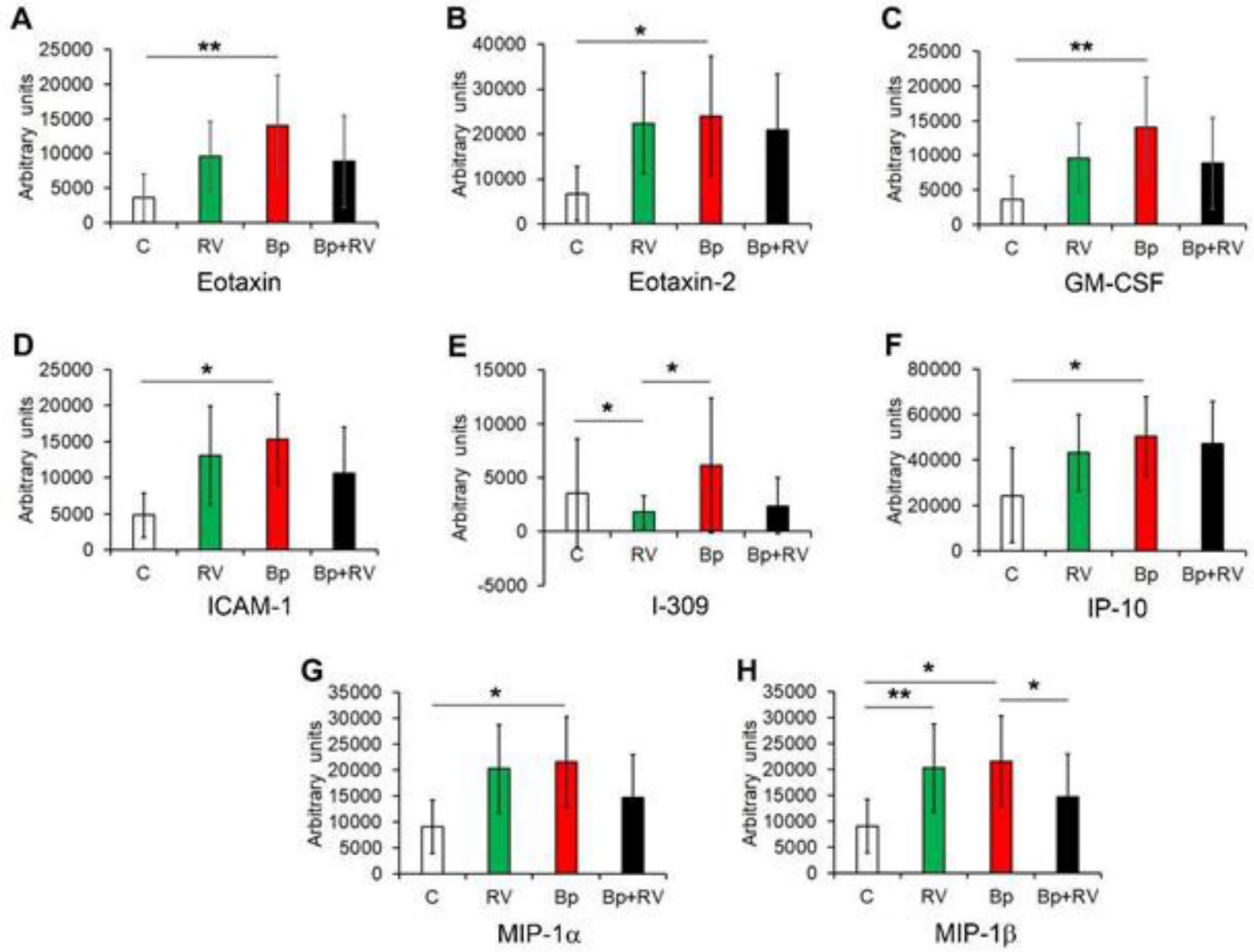
Differentially expressed inflammatory proteins in the screening cohort analysed by antibody array. The histograms reported the mean ± SD of semi-quantitative expression in controls (C), RV patients, Bp patients, and Bp + RV patients of Eotaxin (**A**), Eotaxin-2 (**B**), GM-CSF (**C**), ICAM-1 (**D**), I-309 (**E**), IP-10 (**F**), MIP-1α (**G**), and MIP-1β (**H**). *P < 0.05; **P < 0.01, by ANOVA.

**Figure 5:**
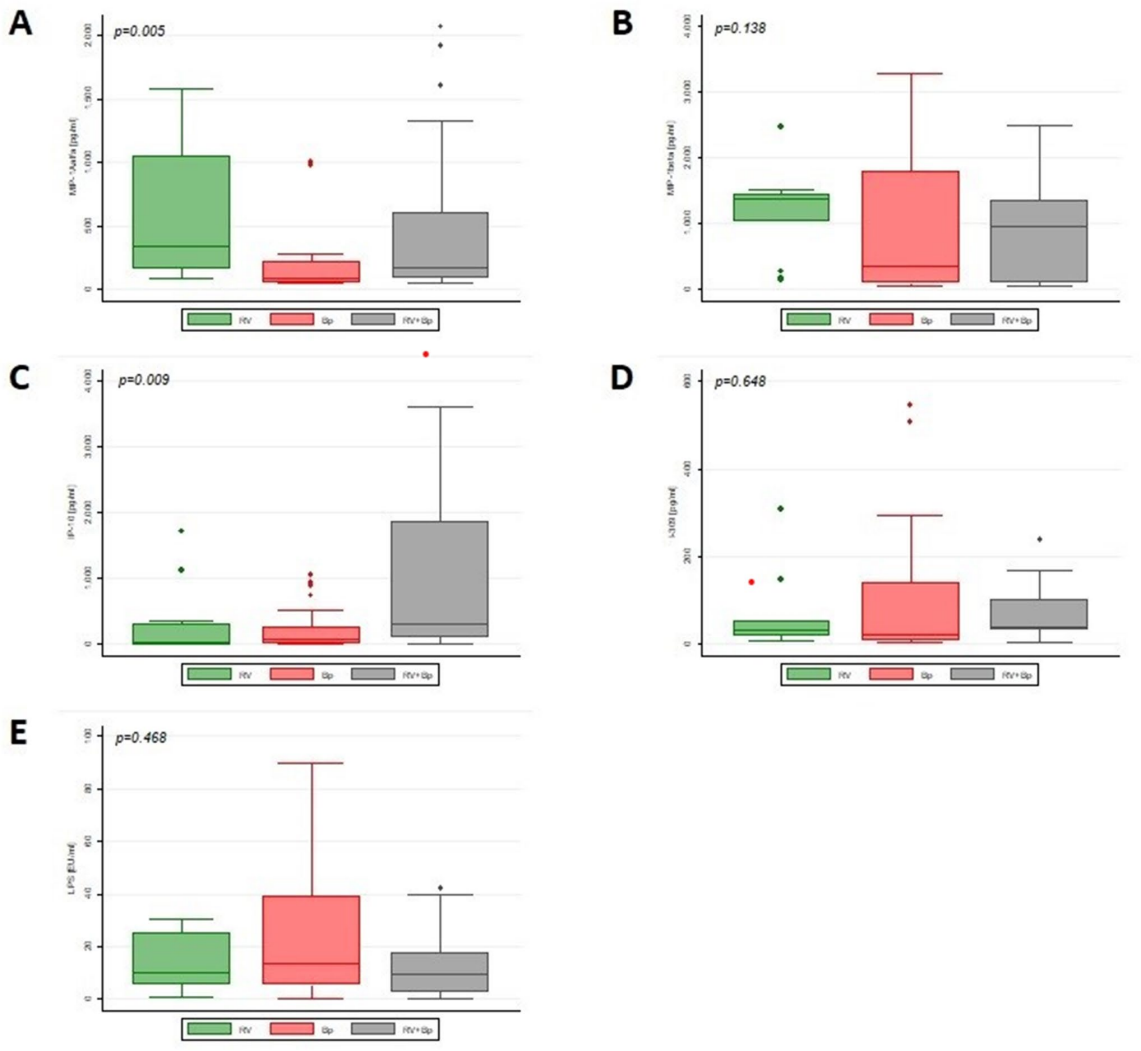
Quantitative changes of specific inflammatory proteins in the validation cohort. The box plots reported the median ± SD of semi-quantitative expression in controls (C), RV patients, Bp patients, and Bp + RV patients of MIP-1α **(A)**, MIP-1β **(B)**, IP-10 **(C)**, I-309 **(D)**, and LPS **(E)**.

The semi-quantitative analysis, which was performed by membrane antibody arrays (Fig. 1B), showed that these inflammatory molecules were differentially expressed among the groups (Fig. 2). In particular, semi-quantitative analysis of membranes revealed that 32 inflammatory molecules showed no statistically significant differences (Fig. 3), while 8 proteins exhibited statistically significant differences among groups (Fig. 4A-H). Among the significantly modulated cytokines, Eotaxin-1, Eotaxin-2, Granulocyte-Macrophage Colony-Stimulating Factor (GM-CSF), Intercellular Adhesion Molecule 1 (ICAM-1), macrophage inflammatory protein-1 β (MIP-1β) and macrophage inflammatory protein-1α (MIP-1α) and IP-10 were significantly up-regulated in Bp group compared to RV group and to control group too. Moreover, as shown in Fig. 5E, I-309 cytokine, also known as C-C motif chemokine ligand 1 (CCL1), was significantly down-regulated in RV group when compared to Bp group and even in comparison to controls. Interestingly, MIP-1β was significantly up-regulated in both RV and Bp group if they were compared to control group, while it was down-regulated in Bp + RV with respect to Bp (Fig. 4H).

### Validation and correlation analysis of inflammatory molecules with patient’s data

Among the inflammatory molecules found to be differentially expressed by array analysis, we validated MIP-1α/β, IP-10 and I-309 by ELISA quantitative analysis in a larger sample of available NAPs from children with Bp, RV infection or co-infection. In total, we analysed 58 children: 14 with RV, 24 with Bp and 20 with Bp + RV. As shown in Fig. 5, MIP-1α levels were significantly up-regulated in RV infection group and co-infections compared to Bp infection group (*P* = 0.005), and IP-10 levels were significantly up-regulated in co-infection group and Bp infection group, compared to RV infection group (*P* = 0.009). No significant change was found in the levels of MIP-1β, I-309, and LPS. Spearman’s coefficient analysis demonstrated that there is no correlation between the levels of the significant cytokines (MIP-1α and IP-10) and sociodemographic and clinical data of patients. Moreover, in the multivariate multinomial logistics model we found that with the increase of IP-10 and in the presence of antibiotics before admission decreases the risk of having a Bp infection compared to the risk of having a co-infection (Table 3).

**Table 3:** Multivariate multinomial logistics model.

Rhinovirus versus Co-infection	OR	95% CI	*P*
Age at symptoms’ onset, months	0.986	0.281–3.455	0.982
Vaginal delivery	0.273	0.013–5.804	0.405
Cough duration, days	0.737	0.517–1.050	0.091
Whooping	1.031	0.355–29.970	0.986
White cell count > max for age	0.020	0.000–3.235	0.132
Antibiotic treatment before admission	0.510	0.006–46.836	0.771
MIP-1alpha	1.001	0.998–1.003	0.637
MIP-1beta	1.002	1.000–1.005	0.097
IP-10	0.998	0.995–1.001	0.120
LPS	1.027	0.921–1.145	0.632

Bordetella versus Co-infection	OR	95% CI	*P*
Age at onset of symptoms, months	0.654	0.314–1.363	0.257
Vaginal delivery	1.649	0.200–13.566	0.642
Cough duration, days	0.956	0.805–1.135	0.605
Whooping	2.908	0.3556–23.784	0.319
White cell count > max for age	1.099	0.102–11.877	0.938
Antibiotic treatment before admission	0.065	0.007–0.578	**0.014**
MIP-1alpha	0.997	0.993–1.000	0.089
MIP-1beta	1.001	0.999–1.002	0.221
IP-10	0.997	0.993–1.000	**0.036**
LPS	1.073	0.995–1.158	0.067

## Discussion

Our findings demonstrated that Bp and RV co-infection is clinically indistinguishable from Bp infection alone, with the exception of a higher use of antibiotics before admission. The socio-demographic characteristics of this sample of patients were similar to patients enrolled in the enhanced pertussis surveillance^19^.

Compared to RV infection, co-infections have a longer cough duration, increased lymphocytes, and white cell count, and more frequently require antibiotic treatment before hospitalisation. Patients with co-infections seem to have a higher immune response compared to single infections and it is more similar to pertussis infection alone than to rhinovirus infection.

Moreover, children with co-infection exhibited different inflammatory profiles in the nasopharyngeal niche with respect to single infections. Most cytokines were the same between groups while some of them were up or downregulated within the three groups. Indeed, levels of IP-10 were higher in NPA from patients with co-infection with single pathogens; whilst MIP-1α levels were significantly up-regulated in the RV infection group compared to the Bp and co-infection groups. Chemokines are the dominant type of cytokines unregulated in the NPA samples from Bp infected children. They are mainly secreted by epithelial cells and their key role is in neutrophil recruitment.

Correlation analysis demonstrated that the trends seen in these specific pro-inflammatory molecules did not align with patterns of clinical manifestation. The lacking of differential clinical patterns could be due to the small sample size, or to as yet undefined mechanisms of interaction between the two pathogens that modulate host response. This result is in line with a previous study where strong differences in clinical features were not found between the three groups^15^.

However, our findings are suggestive of low-grade chronic inflammation in the nasopharyngeal niche, which is the first site of colonisation for bacteria and viruses that cause respiratory disease. IP-10 levels in particular could be used to discriminate single from mixed pathogen infections. Indeed, several studies have reported that IP-10 is associated with the presence of respiratory viruses. IP-10 is a ligand for the CXCR3 receptor, and acts as a chemoattractant for activated Th1 cells, and natural killer cells^20–22^. This inflammatory mediator has been shown to play an important role in the host response to a variety of viral infections including RVs^23^, respiratory syncytial virus^24,25^, herpes simplex virus and hepatitis C virus^26–28^. Previous studies have also demonstrated that IP-10 is released from cultured human airway epithelial cells in response to RV^23^ and H5N1 influenza infection^29^. Moreover, IP-10 was detected in respiratory samples of patients with RV upper airway infection^23^ and RSV bronchiolitis^24^. Finally, recent studies suggest that serum IP-10 level may be an important biomarker for various viral infections and CXCL-10/IP-10, among other chemokines, is also related with mortality rate^30–32^.

Therefore, IP-10 could be relevant as a biomarker of inflammation and outcome in cases of co-infection. Indeed, although we have no relevant data about mortality rate in Bp + RV, other bacterial-viral co-infections have been previously associated with an increased severity and a higher lethality^33^ due to still unknown mechanisms^16,34–39^. Evidence suggests that mechanisms of severe morbidity following viral-bacteria co-infection could include the failure of an antibacterial immune response, pathogen synergy and, failure to resume function and tolerance^40–42^. Bp exploits a broad variety of virulence factors to establish efficient infection. It uses macrophages as an intracellular niche and that the intra macrophage phase of infection could play a significant role in survival and persistence of bacteria within the host^43–45^. Moreover, it has been reported that expression of IP-10 in mixed infections occurred earlier than in single infection possibly promoting a faster and stronger macrophage response^4^.

Further studies on larger populations are needed to confirm our findings and discover their mechanistic role.

This study has several potential limitations. We studied only the association with RV that probably has a milder impact on the clinical course of Bp compared to that of other viruses. Our results are limited to a hospital setting in which we probably could have selected more severe patients and could be not generalizable to the general population. Moreover, we have a small sample size, because we retrospectively studied a subset of patients for whom a leftover of the NPA was available.

The main strength of this study is that we simultaneously screened for Bp and viruses all children presenting with respiratory symptoms, for whom we have clinical and laboratory diagnosis. The etiological diagnosis of respiratory infections is difficult and it is crucial to use as routine, a multiplex RT-PCR for fast and correct identification of the etiological agent^46–48^. The ongoing approach in many hospital settings is to perform a PCR for viruses only when there is a clinical suspicion of an overlap infection. Several studies indicate that co-infection of Bp with a respiratory virus is a common finding when a multiplex PCR is performed and has controversial clinical effects on the course of the disease^46–49^.

Our results need to be confirmed in further studies investigating the role of IP-10 and other inflammatory molecules, as biomarkers and/or potential therapeutic targets against hyper-inflammatory response occurring in mixed infections.

## Materials and methods

### Statement on guidelines

All methods were carried out in accordance with relevant guidelines and regulations.

### Study design, setting and population

An observational study was conducted at Bambino Gesù Children’s Hospital (OPBG), a 600-bed tertiary centre for paediatric care and research in Rome, Italy.

In the context of an enhanced surveillance program, between January 2016 to December 2019, we routinely screened all infants < 1 year of age who presented at the emergency room with symptoms compatible with pertussis according to the ECDC clinical case definition. Screening was conducted for Bp and 16 respiratory viruses (adenovirus, influenza A and B virus, parainfluenza virus 1–2-3–4, metapneumovirus, coronavirus OC43, 229E, NL-63, rhinovirus, bocavirus, enterovirus) using a multiplex RT-PCR on nasopharyngeal aspirate (NPA). A subset of patients with single or mixed infections for whom a leftover of the NPA was available were then selected. The study was approved by the Ethical Committee of Bambino Gesù Children’s Hospital and IRCCS (Roma, Italy). Informed consent was obtained from the parents or legal guardians of all participants.

### Definitions

For the purpose of this study, we considered pertussis cases as those with a Bp positive PCR, a viral infection as those having a positive RT-PCR test for RV only, and co-infections as those who had positive test results simultaneously for both Bp and RV. For diagnostic markers that were indicative of Bp infection: White Blood Cell (WBC) count was considered greater than the maximum value for age if it was above 34,000 for children < 1 month of age and 14,000 for children ≥ 1 month of age^50^. Lymphocyte percent was considered elevated if it was ≥ 50%.

### Data collection

For each enrolled patient, we collected the following data through a questionnaire administered to patients’ parents by the researchers: sociodemographic data, gestational age, type of delivery, birth weight, education-level, parental employment, date of symptom onset, exclusive breastfeeding at symptom onset, number of people in the household, number of smokers in the family. Based on hospital data, we also recorded the following variables: length of hospital stay (date of admission and discharge), diagnosis at admission and at discharge, complications during hospitalisation, and antibiotic treatment used before admission.

### NPA and RT-PCR for Bp and viruses

NPA were collected and processed using a standardised protocol: Samples were collected within 24 h of hospital admission and processed immediately, or stored at -70 °C until performing the test. Nucleic acids were extracted from a 200 μl sample of nasopharyngeal aspirates and purified, using the EZ1 Virus Mini Kit v. 2.0 on the EZ1 Advanced XL platform (Qiagen, GmbH, Hilden, Germany). Nucleic acid extracts were eluted into 90 μl of buffer and processed immediately. The presence of Bordetella was investigated using Bordetella Real Time PCR kits, targeting IS481 (Bordetella R-gene™ assay (Argene, Biomerieux, Marcy l’Etoile, France).

NPA were performed and processed using a specific panel that also detected the following viruses: RSV A and B, influenza virus A and B, human coronavirus OC43, coronavirus 229E, coronavirus NL63, HUK1, adenovirus, RV, parainfluenza virus 1–2–3–4, human metapneumovirus-hMPV and human bocavirus-hBoV.

We also collected spontaneous sputum samples from 6 children (mean age 10 years) who were admitted to Bambino Gesù Children’s Hospital for non-pulmonary illnesses, and we used them as controls to obtain reference values in cytokines’ semi-quantitative analysis, since it was not possible to perform a NPA in healthy children.

### Inflammatory mediators by antibody array and enzyme-linked immunosorbent assay (ELISA)

Sputum and NPA samples were thawed, protease inhibitor cocktail was added (Halt™ Protease Inhibitor Cocktail (100X), Thermo Fisher Scientific Inc.; Waltham, MA, USA), concentrated by centrifugation at 250xg (10 min, 4 °C), and the supernatant was aliquoted and stored at -80 °C for further protein concentration measurements. Protein concentration was measured in each sample using the BCA method in a kit (Pierce BCA Protein Assay Kit, Thermo Fisher Scientific Inc.). Fifty micrograms of proteins were used for the analysis of 40 cytokines using the Human Inflammation Antibody Array-Membrane Kit (ab134003, Abcam, Cambridge, UK) following the manufacturer’s instructions. In brief, the array membranes were blocked by incubation with a blocking buffer for 30 min. After incubation, membranes were incubated with biotin-conjugated anti-cytokines overnight at 4 °C and then with HRP-Conjugated Streptavidin for 2 h. The signal was detected by chemiluminescence with iBright Western Blot Imaging Systems (Thermo Fisher Scientific Inc.). Sample comparison was achieved using densitometry software for a semi-quantitative analysis (Image Studio Lite_5.2.5, LI-COR Biosciences, NE, USA). For specific ELISA, samples were diluted and analysed using commercially available kits according to the manufacturers' recommended protocols. Quantitative determination of MIP-1α, MIP-1β (Macrophage Inflammatory Proteins -1α and -1β) and IP-10 (Interferon gamma-induced protein 10) chemokines was performed by using a Quantikine ELISA kit (R&D Systems, MN, USA), while I-309 levels were measured using the colorimetric human CCL1/I-309/TCA-3 ELISA Kit (Novus Biologicals, CO, USA). Lipopolysaccharide (LPS) concentrations were detected by a LAL (Limulus amebocyte lysate) chromogenic endpoint assay purchased by Hycult Biotech (Uden, The Netherlands). Absorbance was measured with the Infinite F50 microplate reader spectrophotometer from Tecan (Tecan Group Ltd, Männedorf, Switzerland) and data analysis was executed with Magellan v7.0 software (Tecan Group Ltd.).

### Statistics

Socio-demographic and clinical characteristics of patients enrolled in the study were described as means and standard deviations (SD) or proportions. Cytokine distributions were described through box-plots. Differences between proportions of the three groups of patients (positive for rhinovirus, for Bordetella pertussis or for both) were studied through the Chi-square test or Fisher’s exact test when appropriate, while differences between means were studied through the Kruskal–Wallis test. After verifying for an absence of multicollinearity, a multivariate multinomial logistic regression was used to explore the association between the type of infection, considering co-infections as reference, and the independent variables with *P* < 0.20 at univariate analysis, more precisely: age at symptoms onset (months), type of delivery (vaginal vs. caesarean), cough duration (days), whooping, white cell count > max for age , antibiotic treatment before admission, cytokines (MIP-1alpha, MIP-1beta, IP-10, LPS). A *P*-value < 0.05 was considered statistically significant. Stata 13 was used to perform statistical analyses.

### Ethics approval

The study was approved by the Ethical Committee of Bambino Gesù Children’s Hospital and IRCCS (Roma, Italy). Informed consent was obtained from the parents or legal guardians of all participants.

### Consent to participate

Informed consent was obtained from the parents or legal guardians of all participants.

### Consent for publication

Not applicable.

## Data Availability

All data generated or analysed during this study are included in this published.

## Abbreviations

Bp: Bordetella pertussis
RV: Rhinovirus
RSV: Respiratory syncytial virus
NPA: Naso-pharingeal aspirate
RT-PCR: Real Time Polymerase chain reaction
ELISA: Enzyme-linked Immunosorbent assay
GM-CSF: Granulocyte-Macrophage Colony-Stimulating Factor
ICAM-1: Intercellular Adhesion Molecule
IP-10: Interferon gamma-induced protein 10
MIP-1β: Macrophage inflammatory protein-1 β
MIP-1α: Macrophage inflammatory protein-1α
CCL1: C-C motif chemokine ligand 1
